# Spontaneous Necrotizing Soft Tissue Infection and Fatal Clostridium septicum Septicemia in Myelodysplastic Syndrome: A Case Report and Comprehensive Literature Review

**DOI:** 10.7759/cureus.84715

**Published:** 2025-05-23

**Authors:** Ifeoma Achebe, Chioma Nwachukwu, Chukwuemeka Nzewi

**Affiliations:** 1 Internal Medicine, Genesis Health System, Davenport, USA; 2 Internal Medicine, Montefiore Health System, New Rochelle, USA; 3 Internal Medicine, UnityPoint Health, Rock Island, USA

**Keywords:** clostridium septicum, malignancy, myelodysplastic syndrome, necrotizing fasciitis, sepsis

## Abstract

Necrotizing soft tissue infection (NSTI) is a rare but life-threatening disease characterized by rapid spread and necrosis of the skin, subcutaneous tissue, fascia, and muscle. We present a fatal case of atraumatic NSTI involving the left lower extremity in an 80-year-old female with advanced myelodysplastic syndrome. Blood cultures grew Clostridium septicum and Staphylococcus epidermidis, the latter thought to be a blood contaminant. The patient rapidly progressed to septic shock and died within 34 hours of presentation. Our case emphasizes the need for a high index of suspicion for NSTI in patients with advanced malignancies.

## Introduction

Necrotizing soft tissue infection (NSTI) is a rare and highly lethal condition characterized by rapid necrotizing destruction of the skin, subcutaneous tissue, fascia, and muscle [1-4]. Without timely treatment, rapid microbial proliferation and toxin release can cause fulminant systemic inflammation, septic shock, and death [5-8].

Diagnosing NSTI presents a significant challenge, particularly during its initial stages, as it exhibits similarities with common benign conditions such as cellulitis, erysipelas, and gout [2,9,10]. This similarity often results in delayed or missed diagnoses, leading to elevated rates of morbidity and mortality [10]. There is a growing trend of using “NSTI” as a substitute for the original term “necrotizing fasciitis” (NF) to incorporate all necrotizing infections affecting one or more layers of soft tissue components [3-7,11,12]. NSTIs were originally grouped into two categories based on microbiology: polymicrobial and monomicrobial infections (also known as types 1 and 2, respectively) [13]. This initial classification has evolved to encompass other organisms that have been implicated in NSTIs [5]. Type I NF is characterized by polymicrobial infection, typically involving one anaerobic species alongside one or more facultative anaerobic streptococci and members of Enterobacteriaceae [2]. Type II NF is caused by monomicrobial infection, primarily by group A hemolytic streptococci [2]. Type III NF results from marine Vibrio species, while type IV NF is predominantly fungal, commonly attributed to Candida [2].

Clostridial species, frequently found in soil, humans, and animal intestinal tracts, can present in two primary forms: traumatic and spontaneous NSTI [14]. The traumatic subtype is most frequently attributed to Clostridium perfringens, whereas the spontaneous form, often resulting from hematogenous seeding of muscle with bacteria, is typically due to Clostridium septicum [14,15].

Myelodysplastic syndrome (MDS) is a group of hematologic malignant disorders in which the bone marrow does not produce enough healthy cells. Instead, it creates abnormal cells with decreased function, which can lead to anemia, frequent infections, and bleeding problems.

Management of NSTI necessitates a multidisciplinary approach, encompassing prompt administration of intravenous antibiotics and extensive surgical debridement, and may require collaboration among various healthcare providers, including emergency physicians, hospitalist physicians, infectious disease specialists, surgeons (plastic, orthopedic, general surgeon, etc.), and palliative care team [16,17]. With an increasing number of NSTI cases reported in the literature, maintaining a high index of suspicion is crucial for early diagnosis and expedited surgical intervention. Diagnosis may be challenging early in its course as it tends to mimic benign conditions such as cellulitis. The association between C. septicum bacteremia/NSTI and occult or overt solid malignancies, especially colorectal tumors, has been well documented in the literature [18-27]. This report highlights a rare presentation of C. septicum NSTI in an elderly patient with MDS, a hematologic malignancy, an association scarcely reported in existing literature.

## Case presentation

We present a case involving an 80-year-old Caucasian woman with advanced MDS on biweekly transfusion of blood products. Of note, the patient’s MDS was initially treated with chemotherapy, exogenous erythropoietin, and luspatercept without a good response. Her last blood transfusion was a day before she came to our emergency department. Pertinent family history includes breast cancer in the mother and unspecified cancer in the grandmother. The patient was a former smoker (who quit at 25 years of age) and denied alcohol or illicit drug use. She declined a colonoscopy for colon cancer screening but opted for a fecal occult blood test (FOBT), for which she had a negative screen at age 74. However, the subsequent yearly FOBT screening was not pursued.

Her symptoms started around 5:30 am when she was awakened by left ankle, calf, and foot pain, and she presented to the emergency department at 7:34 am the same day. She described the pain as severe, rating it 10/10 in intensity, which was disproportionate to her physical exam findings, exacerbated by weight bearing, plantar flexion, and rotation. She denied any history of trauma, falls, insect bites, recent travels, and blood clots in the past. She denied subjective fevers and chills, rash, weakness, chest pain, and palpitation. Before arriving at the emergency department, she had taken 650 mg of Tylenol and Tramadol without relief. On arrival, she was fully alert and oriented to person, place, time, and situation. Her initial vital signs were as follows: temperature of 97.1°F (tympanic), a blood pressure of 121/64 mmHg, heart rate of 66 bpm, and respiratory rate of 16 breaths per minute with oxygen saturation at 97% on room air. She exhibited significant tenderness in her left distal calf, ankle, and foot upon palpation. The rest of her physical examination was unremarkable, with no erythema, swelling, or rash noted in the area. There were no motor deficits. Peripheral pulses were intact, and the Thompson test was negative for Achilles tendon rupture.

Initial laboratory investigations revealed a normal white cell count (WCC), mild thrombocytopenia, moderate anemia, normal creatinine, normal sodium level, and hyperglycemia (Table 1). X-rays of the left ankle were normal, and venous Doppler ultrasound showed no evidence of deep venous thrombosis. Gout was considered due to an elevated uric acid level.

**Table 1:** Clinical laboratory values and reference ranges

Parameters	Admission	At 16:28	At 05:30 (next day)	Reference range (units)
White Cell Count (WCC)	7.0	18.3	12.9	4.8-10.8 thousand/uL
Hemoglobin (Hb)	8.3	7.6	7.3	12-16 gm/dL
Platelet Count (PC)	110	135	109	130-450 thousand/uL
Sodium, Na	137	-	129	134-144 mmol/L
Glucose, Glu	135	-	197	65-99 mg/dL
Creatinine	0.66	-	0.73	0.76-1.27 mg/dL
Uric acid	7.6	-	-	2.5-7.1 mg/dL
Erythrocyte Sedimentation Rate (ESR)	-	29	48	0.0-30 mm/hr
C-Reactive Protein (CRP)	-	3	17.4	0.0-0.5 mg/dL

She received IV ketorolac 15 mg and IV morphine 2 mg for pain control. Initially, the plan was to discharge her with pain medications, but due to persistent severe pain, she was admitted for further evaluation and pain management.

**Figure 1:**
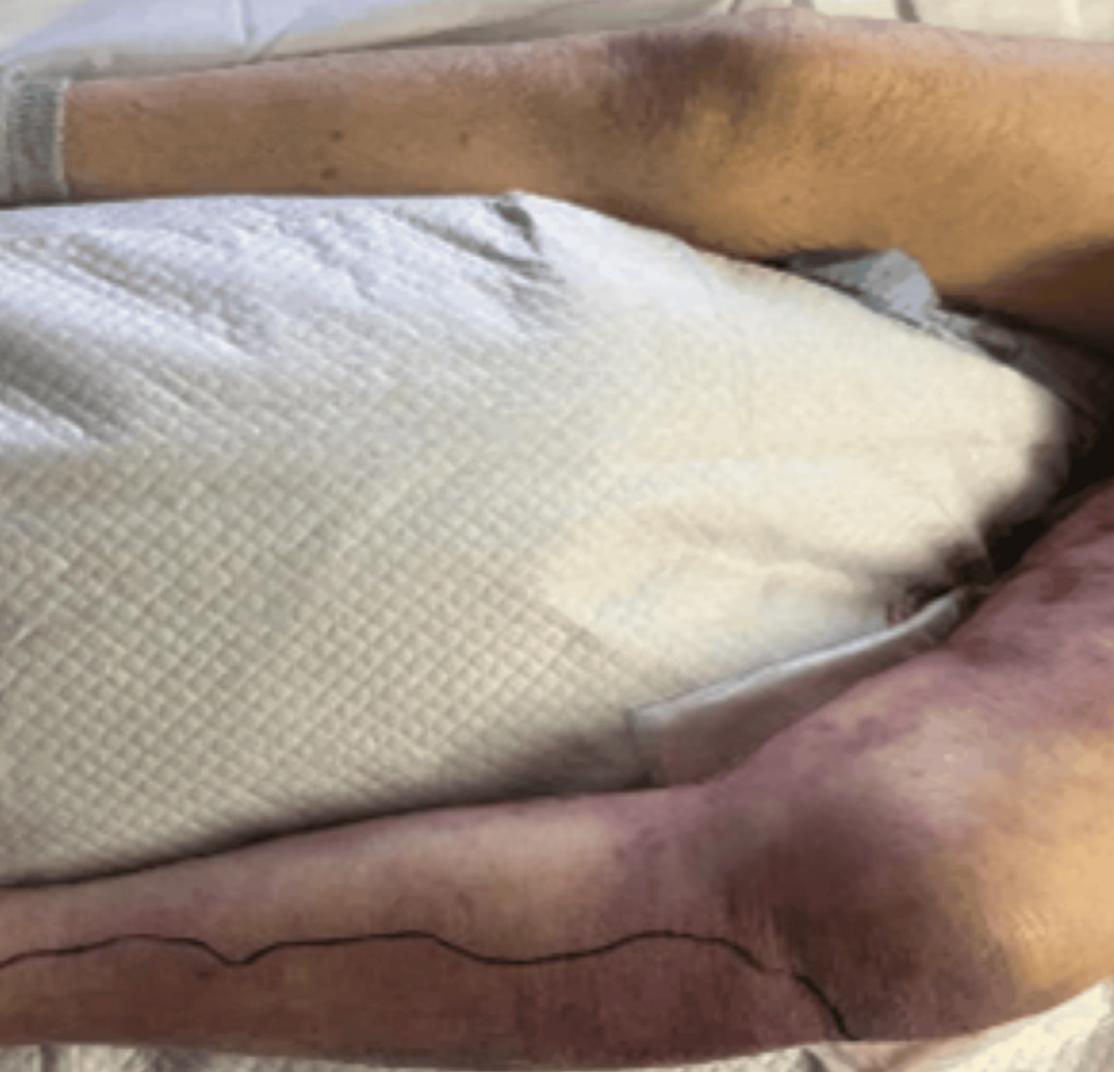
Necrotizing soft tissue infection (NSTI) involving the left lower extremity of an 80-year-old female showing marked extremity swelling and ecchymotic patches in comparison to the normal right lower extremity

**Figure 2:**
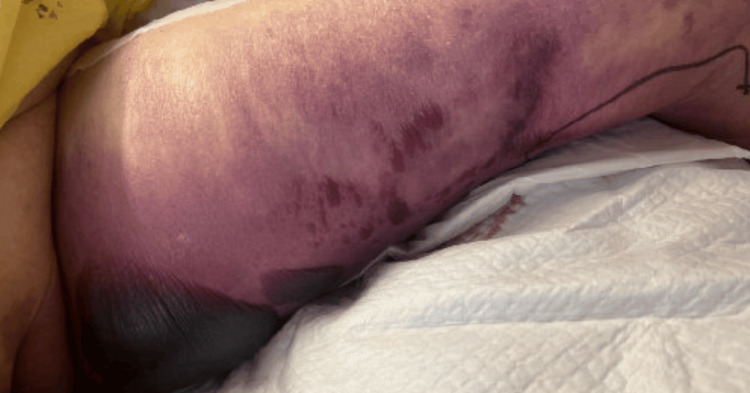
Formation of hemorrhagic bullae in the left thigh

At 16:28, her physical examination revealed mild edema developing in the left lateral malleolus and ecchymosis in the left lateral calf area. Although she remained afebrile, she exhibited tachycardia. Repeat laboratory tests showed leukocytosis, decreased hemoglobin, and increased platelet count from prior. Erythrocyte sedimentation rate (ESR) and C-reactive protein (CRP) were elevated (Table 1). An MRI of the left lower extremity was ordered. Management at this stage included opiate pain control and leg elevation. Overnight, her physical exam findings worsened, with the development of hemorrhagic blisters and increased leg swelling (Figure 1 and Figure 2).

**Figure 3:**
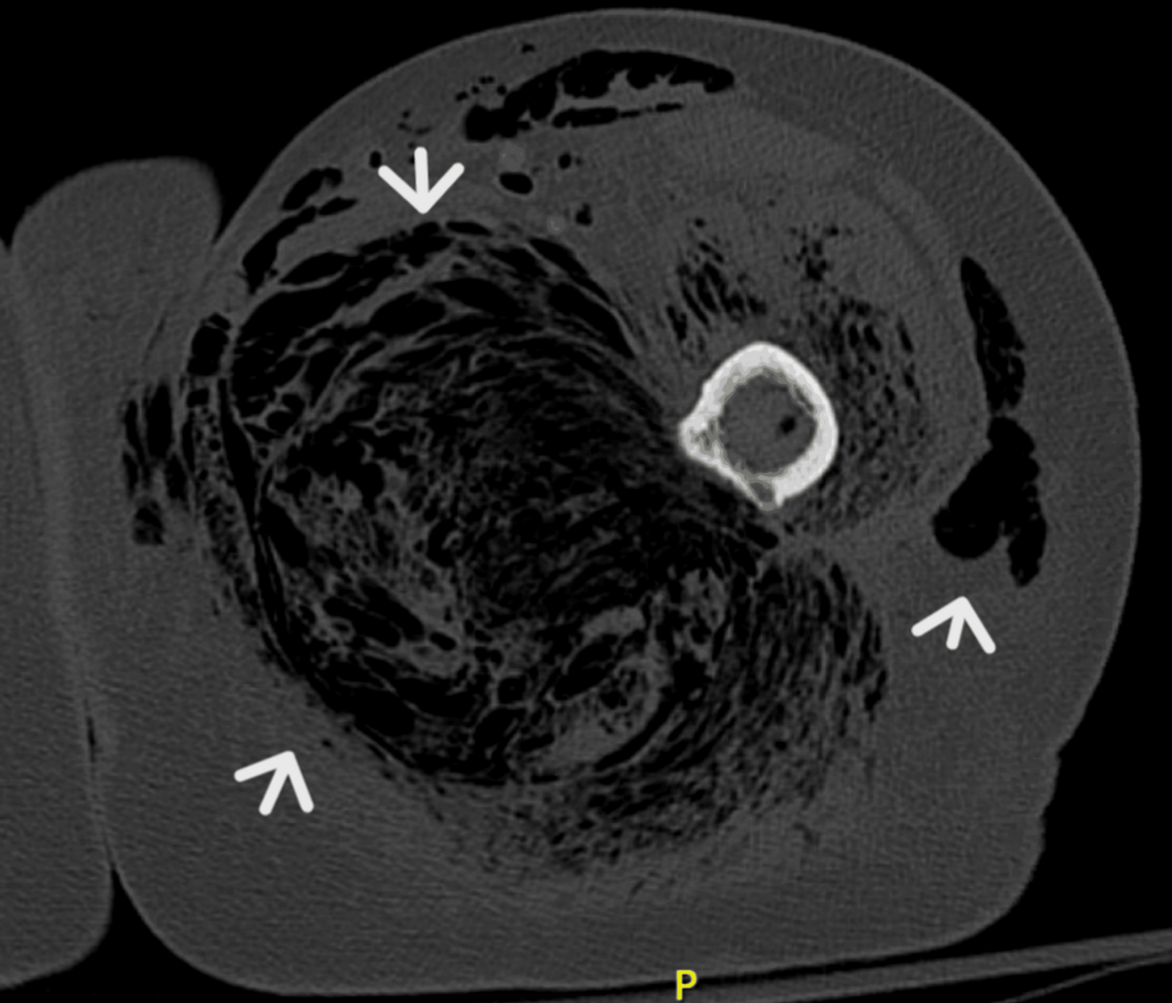
Computerized tomography scan showing air insinuating throughout the subcutaneous fat and muscular planes with air foci present within the bone (arrows)

**Figure 4:**
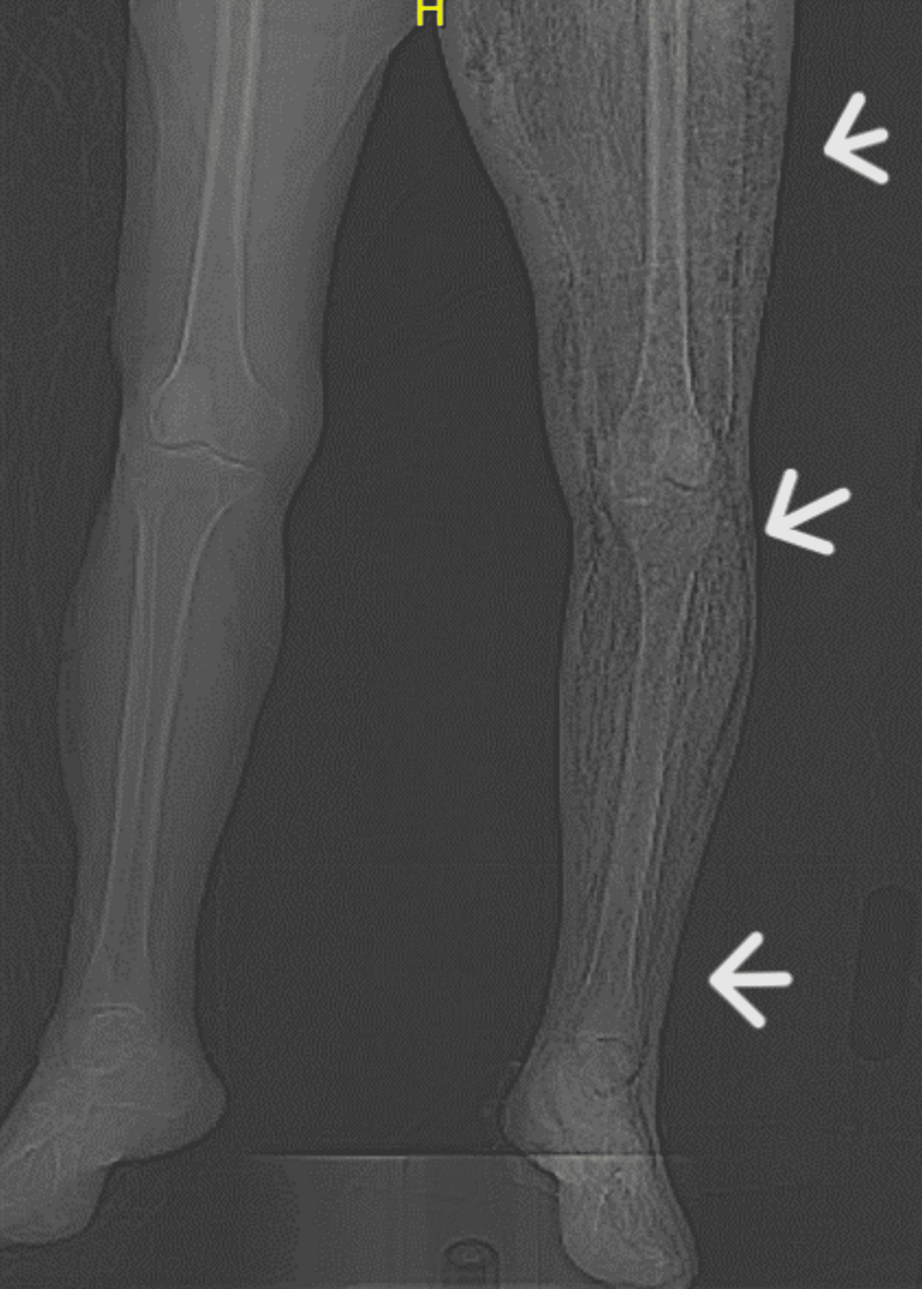
Extensive soft tissue emphysema of the left lower extremity in comparison to the right (arrows)

At 5:30 AM the following day, her left foot appeared pale, and she experienced lower extremity numbness and altered mentation. An urgent CT scan in place of the MRI was performed due to the rapid progression of symptoms, which confirmed extensive NF involving the entire lower limb (Figure 3 and Figure 4). A radiographic diagnosis of severe NF was made. Subsequent laboratory tests showed a further drop in hemoglobin, platelet count, and sodium level, while white blood cell count improved with worsening hyperglycemia, CRP, and ESR (Table 1). The patient's family was informed of the diagnosis, but they were initially undecided on how to proceed with the patient's management. An initial extensive goals of care meeting was conducted.

The patient was eventually commenced on intravenous antibiotics empirically at 14:22 with permission from her family, followed by consultation with the surgical team. The benefits, risks, prognosis, and potential outcomes of an emergency surgical debridement were discussed with the patient’s family. The surgeon recommended transferring the patient to the nearest tertiary facility with adequate tools and expertise for extensive surgical debridement. After careful consideration, the family chose to pursue comfort care ultimately. The palliative team was consulted, and comfort measures were instituted. Prior to formal enrollment with hospice services, the patient rapidly deteriorated with the development of hypoxic respiratory failure, shock, apnea, and subsequent asystole.

**Figure 5:**
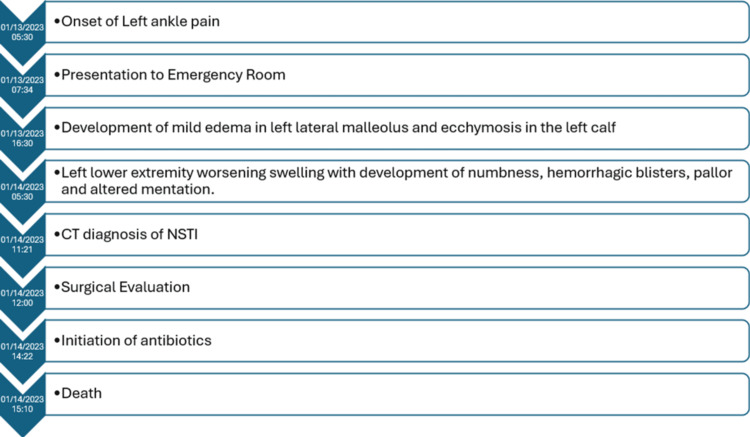
Timeline of key events in the index patient depicting rapid clinical course Timeline showing clinical course and key interventions in the case from symptom onset to the death of the patient.

The patient died at 15:10, approximately 34 hours after the onset of her symptoms (Figure 5) and before the results of her cultures were available. An autopsy was not conducted. Notably, blood cultures revealed C. septicum and Staphylococcus epidermidis in two distinct sets of culture bottles, with the latter organism considered more likely a contaminant.

## Discussion

NSTI is a rare, highly lethal condition characterized by rapid necrotizing destruction of the skin, subcutaneous tissue, fascia, and muscle [1-4]. Every year, about 500-1,500 persons are diagnosed with NSTI in the United States [28], with an average mortality of approximately 25% [5,29]. The risk of case fatality remains highest among the elderly and individuals with comorbidities or immunosuppression [29]. The major challenge in treating people with NSTI often lies in recognizing the disease early in its course, which was evident in our case. This is further complicated by the disease frequently mimicking common non-necrotizing soft tissue infections such as cellulitis or abscesses. In equivocal cases such as our case report, it is clear that relying on initial clinical findings alone may cause a lack of sensitivity in accurately diagnosing NSTIs.

C. septicum has frequently been linked to spontaneous NSTI, in which no visible portal of entry is seen [14,15]. These organisms have been closely linked to occult or overt malignancies, particularly colorectal tumors [18-27]. Pathophysiology is thought to be hematogenous spread, with a primary source likely from the gastrointestinal (GI) tract and resultant seeding of C. septicum in the muscles [14,15].

Notably, this report adds to the limited literature of NSTI cases with C. septicum septicemia in patients with advanced MDS. Interestingly, our case did not undergo a screening colonoscopy but rather had FOBT testing, which resulted in a negative screen for colon cancer. Importantly, an FOBT screen was done on our patient at the age of 74; however, yearly screening, which is the recommendation when using FOBT, was not done. At age 80, she could potentially have an occult malignancy, but this, unfortunately, could not be determined because an autopsy was not done. Our extensive literature search revealed several cases of C. septicum NSTIs associated with GI malignancy or a breach in the GI epithelium from various causes, which makes a strong argument about the GI source of infection [18-27]. These patients did not have a diagnosis of MDS.

Clinical presentation

Patients with NSTI initially have subtle clinical findings, which is why they are often missed or misdiagnosed. The most consistent early presenting symptom is “pain out of proportion to physical examination.” Other symptoms that are encountered include swelling, ecchymosis/discoloration, numbness, crepitus, loss of function, necrosis/gangrene, bullae/blisters, fever, altered mentation, and abnormal breathing. Symptoms tend to rapidly progress over the course of hours to days [2,17,30-36].

Laboratory investigations 

Laboratory Risk Indicator for NF (LRINEC) Score

The LRINEC score was developed by Wong et al. as a clinical tool to aid risk stratification of NF. This tool uses laboratory parameters, such as C-reactive protein (CRP), white cell count (WCC), hemoglobin (Hb), sodium (Na), creatinine (Cr), and glucose [37]. Patients can be classified as either low-risk NF (score < or = 5), medium-risk NF (scores 6-7), or high-risk NF (score > or = 8). In the developmental study, a score of 6 or more resulted in a positive predictive value of 92.0% and a negative predictive value of 96.0% [38]. Usually, a cutoff of LRINEC score > or = 6 is used as an indicator of the need for surgical exploration; it also helps discriminate necrotizing from non-necrotizing soft tissue infection. However, this scoring system should be interpreted with caution, as low scores may falsely reassure clinicians.

In our case, the calculated LRINEC score was 2 on admission (CRP = 0, WCC = 0, Hb = 2, Na = 0, Cr = 0, glucose = 0). Hence, it would be classified under the low-risk category, which would erroneously warrant a more conservative approach. There have been conflicting reports in the literature about the clinical usefulness of the LRINEC score.

Unfortunately, the available studies assessing the LRINEC score use during our literature review had several pitfalls. These include small sample size, which makes it underpowered to show statistical significance; retrospective nature; single-center; incomplete or missing values; heterogenous population studied hence difficult to generalize; over-reliance; and high scoring of CRP, which is nonspecific and can be elevated in acute non-necrotizing conditions, such as cellulitis and chronic inflammatory conditions.

A retrospective study conducted over a period of 10 years in the emergency department concluded that the LRINEC score may not be an accurate tool for NF risk stratification due to the high false-positive rate in detecting NF, which could lead to misleading differential diagnosis, more rigorous workup, and potentially unnecessary invasive intervention [9]. This study also showed that the LRINEC tool accurately assessed NF risk among diabetic patients compared to non-diabetic patients at rates of 74.2% and 43.8%, respectively [9].

Another retrospective study challenged the use of a cutoff > or = 6 to risk-stratify. It showed that, even though NF's sensitivity at cutoff > or = 6 was 80%, its specificity was only 57% [38].

Other uses of this scoring system, particularly in prognostication, have been evaluated in studies. A retrospective study by Tessler et al. revealed that pre-LRINEC scores were associated with escalation in intraoperative care (e.g., arterial line placement, longer anesthesia, and operative times) in patients with NF [39].

A study by Cui et al. showed that non-survival was associated with older age, higher blood urea nitrogen (BUN) or Cr concentration, coagulation disorder, lower sodium concentration, and longer duration from admission to first operation [40]. In our case, the patient is elderly, and the diagnosis of NF was missed, which delayed intervention.

During our literature review, we found a paucity of prospective studies validating the LRINEC score. A prospective study by Hsiao et al. showed that the LRINEC score might be inaccurate in risk-stratifying NF in the emergency setting; hence, this tool should be interpreted with caution [41].

The gold standard for clinically suspected NF, despite a low LRINEC score, remains surgical exploration [2,17].

Cytokines

A prospective multicenter study by Hansen et al., which observed baseline IL-6 levels in patients with NSTI and LRINEC score <6 versus > or = 6, showed cytokine levels were not significantly different in the two risk categories. Rather, elevated cytokine levels correlated more with the severity of infection. Higher cytokine levels were seen in patients with septic shock or high Simplified Acute Physiology Score II (SAPS II) and Sequential Organ Failure Assessment (SOFA) scores. Increased levels also correlate more with renal replacement therapy, amputation, and increased 80-day mortality. IL1B and IL-10 levels had the strongest association with 30-day mortality [42]. However, these are not yet widely used in routine practice.

Procalcitonin

A retrospective study by Kishino et al. explored the usefulness of serum procalcitonin in early discrimination of NSTI and non-NSTI and found that levels of procalcitonin were significantly higher in NF cases compared to cellulitis case studies. This study was, however, limited by a small sample size [43].

Pentatraxin 3

A prospective observational study by Hansen et al., conducted in the intensive care unit setting at Copenhagen University Hospital, a national center for NSTI management, explored the prognostic use of pentraxin 3 (PTX 3) in NSTIs. High PTX 3 was associated with septic shock, amputation, and the risk of death in patients with NSTI [44].

Imaging 

X-Ray

Imaging studies have proven valuable in identifying and evaluating the extent of NSTIs. Gas in the subcutaneous tissue and along the fascial planes on X-ray is highly suggestive of NF, although this is rarely seen in the early stage of the disease [45,46], as in our patient. It is also important to note that soft tissue emphysema is caused by gas-forming organisms; therefore, its absence on X-ray does not rule out NSTI [46]. Additionally, plain X-ray films can help identify potential causes of soft tissue inflammation, such as fractures, joint arthritic changes, hidden foreign bodies, etc. [46,47]. 

Ultrasound

Ultrasound is widely available and gives a quick assessment of the soft tissues. It can differentiate alternative causes, such as abscesses, and guide diagnostic fluid aspiration [48]. Castleberg et al. [10] illustrated a case of a 44-year-old woman with an inflamed left groin and thigh and a fever; her LRINEC score was 6, and a rapid bedside US revealed features suggestive of NF. Sonographic findings of subcutaneous thickening, air, and fluid collection within fascia are consistent with NF [49]. Additionally, an ultrasound can reveal the depth of tissue involvement, which could differentiate deep NSTI from simple cellulitis [50]. In our case report, the ultrasound venous doppler was performed to rule out deep venous thrombosis as the cause of the patient’s symptoms. It could be argued that the Doppler sonogram focused solely on evaluating the lower extremity venous system and not the surrounding soft tissues, which could have been more revealing then. Though less sensitive than the CT or MRI, the ultrasound is time-saving and may be useful in rapid soft tissue evaluation, even in unstable patients. However, the sensitivity of ultrasound in identifying NSTI is variable depending on the affected body part. Ultrasound technology is also highly observer-dependent [51-53].

Computerized Tomography Scan (CT Scan)

The CT scan is considered the foremost imaging technique in evaluating NF, especially given its superior spatial resolution compared to ultrasound or plain X-ray films [47]. The estimated sensitivity of CT in identifying NF is about 80%, but it lacks specificity [54]. CT imaging may reveal increased attenuation in the subcutaneous fat, fascial thickening, and gas within the soft tissues [55-57]. According to a study by Ballard et al. [58], CT imaging in NSTI has good interobserver reliability, especially in identifying the presence of fascial air. CT imaging was pivotal in our case in helping make a timely diagnosis of NSTI when MRI was not feasible.

Magnetic Resonance Imaging (MRI)

MRI, on the other hand, is considered the gold standard for diagnosing NF due to its high sensitivity and specificity, remarkable soft tissue resolution, and characterization [58-60]. An MRI finding of deep intermuscular fascial thickening (>3 mm), extensive tissue necrosis, and diffuse subcutaneous edema typically suggests NF; these features are used to distinguish NF from soft tissue infections such as cellulitis and myositis [59-61]. Franzen et al. reported an unusual case of bilateral painful periorbital edema, which was initially misdiagnosed as acquired angioedema; it later turned out to be NF, as confirmed with an MRI [62]. Although MRI is the gold standard for NF diagnosis, it is expensive, time-consuming, and not suitable for critically ill or hemodynamically unstable patients. This limitation was evident in our case, where the patient became unstable and an emergent CT scan was done instead of an MRI.

Cultures

It is crucial to recognize that bacteremia and sepsis are frequently associated with poor patient prognosis and increased risk of mortality [63,64], which is evident in our case with Clostridium septicemia. Timely collection of blood cultures and abscess, wound, or tissue cultures, whenever feasible, is essential to direct appropriate antibiotic treatment [40,65,66]. However, some organisms, such as Clostridium species, pose a challenge in terms of isolation in culture; thus, an absence of these species in blood cultures cannot reliably exclude systemic infection [63]. Moreover, when interpreting rapid wound or exudate smears, non-pathogenic organisms with similar Gram staining characteristics may pose a challenge because they are not actual infections but are wound colonizers [40,65,66]. Given that NSTIs are often polymicrobial, it is recommended to initiate antibiotic therapy with a broad-spectrum regimen covering Gram positives, Gram negatives, and anaerobic organisms [67]. Subsequently, antimicrobial treatment can be tailored to culture results. In our case, there was a delay in initiating empiric broad-spectrum antibiotics due to an initial diagnostic challenge, and this led to a poor outcome.

Surgical exploration

The definitive diagnosis of NSTI is made via surgical exploration of the affected area in the operating room in a setting of high clinical suspicion [5,63]. In equivocal cases, small incisions with local tissue exploration at the bedside or intraoperative frozen section biopsy can aid in the diagnosis of NSTI [17]. Typically, in NF, the diseased fascia appears edematous and loses its adherence to surrounding soft tissue layers, allowing easy movement of the surgeon’s finger along the fascial plane, the so-called “positive finger sign” [5,63]. While a tissue biopsy can be performed, confirming the NF diagnosis is unnecessary, partly because it may yield false-negative results due to sampling error. The key histopathologic features of NF consist of extensive tissue destruction, vascular thrombosis, bacterial spread along fascial planes, and infiltration of acute inflammatory cells [68,69]. In our case, a tissue biopsy was not done because the patient and family opted for comfort care.

Miscellaneous

Spectroscopy

A prospective study by Wang et al. involving 240 patients with cellulitis and NF revealed that monitoring tissue oxygen saturation using near-infrared spectroscopy could potentially aid in early noninvasive identification of necrotizing tissues [70]. At a cutoff value of tissue oxygen saturation of 70%, the test showed a sensitivity of 100%, a specificity of 97%, and an accuracy of 97% in this study [17,70]. A challenge would be the universal routine implementation of this study in hospitals due to its capital-intensive nature, and it may be best suited in larger hospitals or research institutions.

Molecular Studies

In surgically confirmed NF where cultures yield negative results, polymerase chain reaction (PCR)-based detection of pathogens has proven beneficial in affirming the diagnosis. Kopliku et al. proposed employing the alpha-toxin gene (CSA) real-time PCR, a highly sensitive stool screening assay for detecting C. septicum in individuals predisposed to developing NF. Although further investigation within a larger patient cohort is warranted, their findings indicate that C. septicum is not a typical commensal organism but an opportunistic pathogen. They underscored the necessity for additional research to identify predisposing factors that promote transient carriage of this pathogen, thereby facilitating its potential utilization as a screening tool in high-risk populations [71]. This is not a widely applied screening in routine practice.

Treatment

Early diagnosis is critical to managing NSTIs and is most achievable with a multidisciplinary team approach. Effective NSTI treatment necessitates collaboration between the medical and surgical care teams. Numerous studies [4,58,72-77] have demonstrated that immediate surgical debridement and early initiation of broad-spectrum intravenous antimicrobials are the mainstays of treatment of NSTIs. Other documented adjunctive therapies include hyperbaric oxygen therapy (HBOT), negative pressure wound therapy (NPWT), and intravenous immunoglobulins (IVIG). Due to the absence of specific symptoms consistent with NSTI and documented high mortality associated with delayed debridement, Dapunt et al. suggested in 2013 that a surgeon for operative exploration should immediately assess suspect patients. This agrees with a retrospective single-center review, which revealed that a surgical delay exceeding 24 hours was associated with increased mortality [78]. Early surgical debridement is thought to improve survival via mechanisms such as controlling the source of infection [58,59], collecting tissue for cultures, and enhancing tissue oxygenation, increasing antibiotic efficacy [73]. Many patients frequently require more than one surgical debridement to ensure complete removal of necrotic tissue [4,74,77]. Since most NSTIs are polymicrobial, broad-spectrum intravenous antibiotics should be initiated early while awaiting blood and tissue culture results [4,58,59,73,74,77,79]. Several studies have recommended initial coverage for gram positives, negatives, and anaerobes [58,59,73,76], considering local resistance patterns in the index population [73]. Subsequently, antibiotics are narrowed based on culture reports and sensitivities. Our case, unfortunately, highlights the poor outcome that is expected with diagnostic and treatment delay.

Negative Pressure Wound Therapy (NPWT)

As many patients with NSTI need extensive or repeated debridement or sometimes lifesaving limb amputation, wound care is considered an integral part of treatment. Wound management options post-debridement can be vacuum-assisted closure (VAC)/ NPWT, standard wound care, and reconstructive procedures, such as skin grafting, coverage with flaps, etc. [58,59,74]. Among the benefits of NPWT are exudate removal, decreased toxin absorption, edema resolution, improved local blood circulation, and growth of granulation tissue, which ultimately facilitate wound healing [58]. Several studies and case reports have illustrated the advantages of NSTI management involving vacuum-assisted wound closure [4,59,80-84]. Additionally, although there is a knowledge gap in the literature regarding reconstructive procedures following NF debridement, the plastic surgeon must perform a method most suitable for each patient’s skin defect. lacovelli et al.'s multicenter cohort study indicated benefits in cumulative wound closure rates at 10 weeks and overall survival at 90 days post-initial surgery. In comparison to conventional wound dressings, Huang et al.'s investigation suggested that, while wound VAC therapy incurred higher costs, it potentially offered greater effectiveness and was associated with lower mortality rates in NF patients [85-87].

Hyperbaric Oxygen Therapy (HBOT)

HBOT has been utilized as a supplementary treatment approach alongside surgery and intravenous antibiotics, but its effectiveness remains controversial [73,74]. HBOT functions within the NF management framework by mitigating tissue hypoxia, enhancing intravenous antibiotics' efficacy, and promoting wound healing. It is also thought to eradicate anaerobic pathogens and suppress the synthesis of inflammatory cytokines by increasing blood oxygen levels [58]. Despite the potential advantages, the efficacy of HBOT in NF remains contentious, with conflicting reports. Most investigations into its utility are retrospective, often restricted to healthcare facilities equipped with HBOT capabilities, thus introducing selection bias. Consequently, there remains a pressing need for robust clinical trials to delineate the precise role of HBOT. In scenarios where aerobic organisms predominate in NF pathogenesis, the efficacy of HBOT may be limited [17,53,88-92]. Marongiu et al. reported a unique case of NF of the breast that was successfully treated using a combination of HBOT and NPWT as therapeutic adjuncts [80]. There are additional documented reports of an overall reduction in NSTI morbidity and mortality linked to HBOT [93,94]. However, there remains a lack of data emanating from randomized controlled trials.

Intravenous Immunoglobulin (IVIG)

Like hyperbaric oxygen, IVIG has been suggested as an adjunct therapeutic option, but its role is still uncertain [73,74] with conflicting reports. Research into the role of immunoglobulin in NF management remains scant, with limited evidence suggesting negligible impacts on mortality rates or hospital length of stay. Further comprehensive studies are warranted to elucidate its potential therapeutic benefits [17,53,95]. Darenberg et al. [96] commenced a multicenter, randomized, double-blind, placebo-controlled clinical trial to investigate IVIG's efficacy and safety profile in the treatment of streptococcal toxic shock syndrome. Though the trial was cut short due to poor patient recruitment, available data demonstrated a 3.6-fold increased mortality in the placebo group compared to the treatment group [96]. This may provide some support for the use of IVIG in the management of type II NSTI complicated by streptococcal toxic shock syndrome [73]. Table 2 presents a summary of key literature supporting diagnostic and management challenges in necrotizing soft tissue infections.

**Table 2:** Summary of key literature supporting diagnostic and management challenges in necrotizing soft rissue infections

Study/Author	Year	Design/Setting	Population studied	Key findings	Relevance to the current case
Group 1: Clostridium septicum Infections and Malignancy Associations					
Stevens et al.[15]	1990	Literature review	Cases of spontaneous C. septicum gas gangrene	C. septicum hematogenous spread linked to malignancy	Explains the possible GI source in the patient
Nanjappa et al. [18]	2015	Case report	Patient with colon cancer and C. septicum infection	C. septicum linked to colon malignancy	Supports suspicion of occult malignancy
Mirza et al. [19]	2009	Case report	Patient with spontaneous C. septicum infection	C. septicum infection triggers a malignancy search	Reinforces cancer evaluation despite negative FOBT
Kornbluth et al.[20]	1989	Multicenter retrospective review	Patients with C. septicum infections	High malignancy association	Historical validation of clinical suspicion
Kopliku et al.[71]	2015	Prospective Cross-Sectional Study	Healthy adults	Low prevalence of C. septicum carriage	Validates C. septicum pathogenicity
Group 2: Diagnostic Tools and Laboratory Scores					
Neeki et al.[9]	2017	Retrospective	ED patients with cellulitis or NF	LRINEC has high false-positive rates	Supports cautious use of LRINEC in ED settings
Wong et al.[37]	2004	Retrospective validation	Patient’s with suspected NF	Developed LRINEC score, high predictive value when ≥6, CRP plays a major role	Our case had an LRINEC of 2 but deteriorated rapidly, highlighting its limitations
Holland et al.[38]	2009	Retrospective	Hospitalized with an NF diagnosis	Shows that even though NF sensitivity at cutoff ≥ 6 was 80%, its specificity was only 57%	-
Tessler et al.[39]	2019	Retrospective study, single center	Patients undergoing surgery for NF	Higher LRINEC linked to anesthesia escalation.	Suggests early scoring can identify high-resource cases like ours
Cui et al.[40]	2021	Retrospective	NF patients	Nonsurvivors had high BUN/Cr, low Na, coagulation issues, delayed surgery	Our patient had elevated Cr and a delay in surgery, parallel findings
Hsiao et al. [41]	2020	Prospective validation study	Patients with suspected extremity NF in the ED	LRINEC inaccurate in an emergency setting	Supports cautious use of LRINEC in ED, especially in elderly or atypical cases
Hansen et al.[42]	2017	Prospective Multicenter	NSTI patients with cytokine profiles	Higher IL-6, IL-1B, and IL-10 levels in severe cases; predicted mortality	Reinforces cytokines as potential prognostic markers
Kishino et al.[43]	2021	Retrospective	NF and cellulitis patients	Higher procalcitonin levels in NF patients than in cellulitis	Supports the use of procalcitonin when LRINEC is low or unclear
Hansen et al. [44]	2016	Prospective Observational Study	NSTI patients admitted to the National ICU center (Denmark)	High Pentraxin-3 predicts poor outcomes	Potential biomarker used in NF assessment
Group 3: Imaging Studies and Early Diagnosis					
Castleberg et al.[10]	2014	Case report	Groin/perineal NF	Bedside ultrasound rapidly diagnosed NF	Our case had a negative ultrasound but the study recognizes that ultrasound plays an adjunctive role in NF diagnosis; other imaging modalities can be pursued if clinical suspicion remains high
Wysoki et al.[54]	1997	Retrospective Radiology Review	Hospitalized patients with suspected NF	CT findings correlate with NF diagnosis	CT scan crucial in NF diagnosis
Ballard et al.[58]	2018	Retrospective multicenter radiology study	Patients with suspected NF	High interobserver CT reliability for fascial gas	CT confirmation solid
Franzen et al.[62]	2013	Case report	Misdiagnosed periorbital edema	Misdiagnosed angioedema turned out to be NF, confirmed by MRI	Mirrors diagnostic delay in our case highlights the importance of imaging and suspicion
Wang et al. [70]	2004	Prospective study	Hospitalized patients with NF or cellulitis	Near-infrared spectroscopy had high accuracy	Emerging tool for rapid NF detection
Group 4: Surgical Timing and Outcomes					
Faraklas et al.[29]	2016	Retrospective single-centre review	Patients with NF	Surgical delay worsened survival	Timeliness critical
Dapunt et al. [74]	2013	Literature review + retrospective review	NF patients undergoing surgery	Delay >24h increases mortality	Matches our patient’s delayed outcome
Group 5: Adjunct Therapies					
Marongui et al.[80]	2017	Case report	NF involving the breast	Successful use of HBOT+NPWT; Improved survival	Supports adjunctive therapies in complex NF cases like ours
Huang et al. [85]	2006	Prospective randomized study	Patients with limb necrotizing fasciitis (n=24)	Wound size reduction was seen in 47% (VAC) versus 41% (control), wound VAC therapy incurred a higher cost	-
lacovelli et al. [87]	2020	Retrospective multi-center cohort study	Patients with local versus disseminated Fournier’s disease	VAC therapy improved 10-week wound closure in disseminated cases	-
Darenberg et al.[96]	2003	Multicentre randomized controlled trial (terminated early)	Streptococcal toxic shock patients	IVIG reduced mortality in toxic shock syndrome	Suggests role for IVIG in toxin-mediated NSTIs

Palliative Care/Hospice

NSTI can lead to rapid deterioration and severe pain. In our case, the patient’s family chose comfort care after carefully considering all treatment options and the general prognosis. This decision was respected, and the patient was comfortable until she passed. Establishing a rapid clinical diagnosis is crucial for timely intervention. Discussing treatment goals with patient and their families is essential to ensure they understand their options, including palliative or hospice care, if aggressive surgical options are not feasible or desired. This approach aims to optimize the patient's quality of life and provide appropriate care aligned with their preferences [90].

Strengths and limitations

Our case study provides insight into C. septicum NSTI as a life-threatening disease, its rare association with MDS (a hematological disorder), the potential diagnostic challenges encountered by clinicians, and the need to maintain a high level of suspicion in high-risk patients. C. septicum has typically been implicated in spontaneous NSTI among patients with solid tumors such as colorectal cancer. To our knowledge, there are a few existing reports of C. septicum bacteremia and NSTI in patients with hematological malignancies such as MDS. Our case adds to the handful of literature reporting this association. It highlights diagnostic complexities and delays that clinicians may encounter and how these impact patient outcomes. Discussing NSTI mechanisms, clinical assessment, the workup, treatment options, and lessons learned from the index patient undoubtedly adds educational value to clinicians, researchers, and other knowledge users. The absence of tissue biopsy and autopsy limits definitive confirmation of the diagnosis. Nevertheless, a tissue biopsy may not be considered necessary to confirm NF diagnosis in our patient. CT findings were in keeping with severe NF, and this diagnosis subsequently became clinically evident during gross limb inspection. With regards to culture, specimens have been reported to yield causative organism(s) in only 20-30% of cases, as tissue bacterial burden is typically low in soft tissue infections [97-100]. Although no tissue aspirate or biopsy was taken for cultures, blood cultures were positive for C. septicum, and it is reasonable to presume that C. septicum isolated in the blood seeded the soft tissue, resulting in left lower extremity NF evident on CT imaging. Finally, it is recommended that empiric antibiotics for NSTI cover Gram positives, Gram negatives, anaerobes, and methicillin-resistant Staphylococcus aureus (MRSA), plus an antitoxin agent such as clindamycin. This was not instituted in our case due to the initial diagnostic challenge. It should be noted that our patient stands alone as a single case; hence, we cannot extend all our observations during her hospital course to the wider cancer population. Our patient did not want aggressive interventions and opted for comfort measures. The patient and family’s wishes were respected.

## Conclusions

This case underscores the diagnostic and management complexities inherent in NSTIs, particularly in the context of advanced MDS, a rarity in reported cases. Despite initial clinical indicators suggestive of milder conditions such as cellulitis or gout, the rapid symptom progression calls for a high index of suspicion and timely NSTI intervention. While laboratory investigations such as the LRINEC score, cytokines, procalcitonin, and PTX 3 offer diagnostic potential, their limitations necessitate further evaluation through robust prospective studies. Imaging techniques such as CT scans, MRI, and culture analyses are pivotal in guiding treatment strategies. Emerging technologies, such as near-infrared spectroscopy and PCR-based assays, show promise for early diagnosis but require additional validation through research endeavors. Early surgical exploration and initiation of broad-spectrum antibiotics are key to successfully managing NSTI. These were not instituted early in our case due to the initial diagnostic challenge, which led to a poor outcome. The potential adjunctive roles of therapies such as HBOT and IVIG warrant thorough investigation. Importantly, in cases where aggressive surgical intervention is not feasible or preferred, early discussions regarding palliative or hospice care are indispensable to ensure patient-centered care. Maintaining a multidisciplinary approach and vigilance for NSTIs can influence patient outcomes and quality of life.
